# Repeated complete atrioventricular block during remifentanil administration in a pediatric patient with brain tumor and acute hydrocephalus: a case report

**DOI:** 10.1186/s12871-024-02593-8

**Published:** 2024-08-09

**Authors:** Akihiro Ura, Keisuke Fujii, Tadashi Tanioku, Tomoyuki Kawamata

**Affiliations:** Department of Anesthesiology, Wakayama Medical University, 811-1, Kimiidera, Wakayama-shi, Wakayama, 641-0012 Japan

**Keywords:** Remifentanil, Complete atrioventricular block, Brainstem compression, Pediatric neurosurgery

## Abstract

**Background:**

Remifentanil, an ultra-short-acting µ-opioid receptor agonist, is commonly used for anesthetic management due to excellent adjustability. Remifentanil is known to cause sinus bradycardia, however, because it has a direct negative chronotropic effect on the cardiac conduction system and there is an indirect negative chronotropic effect via the parasympathetic nervous system.

**Case presentation:**

An 8-year-old Japanese boy was diagnosed with acute hydrocephalus due to a brain tumor in the fourth ventricle and underwent emergency surgery. Imaging examination showed brainstem compression. Endoscopic third ventriculostomy and ventriculoperitoneal shunt surgery were scheduled. Remifentanil was started during induction of general anesthesia, but electrocardiogram showed sinus bradycardia, then Wenckebach-type atrioventricular block, and then complete atrioventricular block. Remifentanil was immediately discontinued, and we administered atropine sulfate. Complete atrioventricular block was restored to sinus rhythm. When remifentanil was restarted, however, the electrocardiogram again showed sinus bradycardia, Wenckebach-type atrioventricular block, and then complete atrioventricular block. Remifentanil was again immediately discontinued, we administered adrenaline, and then complete atrioventricular block was restored to sinus rhythm. Fentanyl was used instead of remifentanil with continuous infusion of dopamine. There has since been no further occurrence of complete atrioventricular block.

**Conclusions:**

This is the first known case of complete atrioventricular block in a pediatric patient with increased intracranial pressure seemingly caused by administration of remifentanil.

## Background

Remifentanil, an ultra-short-acting µ-opioid receptor agonist, is commonly used for anesthetic management due to excellent adjustability [1]. Remifentanil is known to cause sinus bradycardia, however, because it has a direct negative chronotropic effect on the cardiac conduction system and there is an indirect negative chronotropic effect via the parasympathetic nervous system [2, 3]. Here, we report a case of a pediatric patient with acute hydrocephalus who developed reproducible complete atrioventricular block after administration of remifentanil. This is the first known report of complete atrioventricular black in a pediatric patient as a result of administration of remifentanil.

Consent was obtained from the patient’s mother for this study.

## Case presentation

An 8-year-old Japanese boy (112 cm, 18 kg) suddenly vomited repeatedly and was brought to our emergency department. At 1 and 2 years of age he had undergone tumor resection surgery for fourth ventricle cerebellar low-grade glioma. At 6 years of age there was tumor recurrence, so he subsequently underwent third ventriculostomy for hydrocephalus. He then received chemotherapy in the pediatric department of our hospital.

**Fig. 1:**
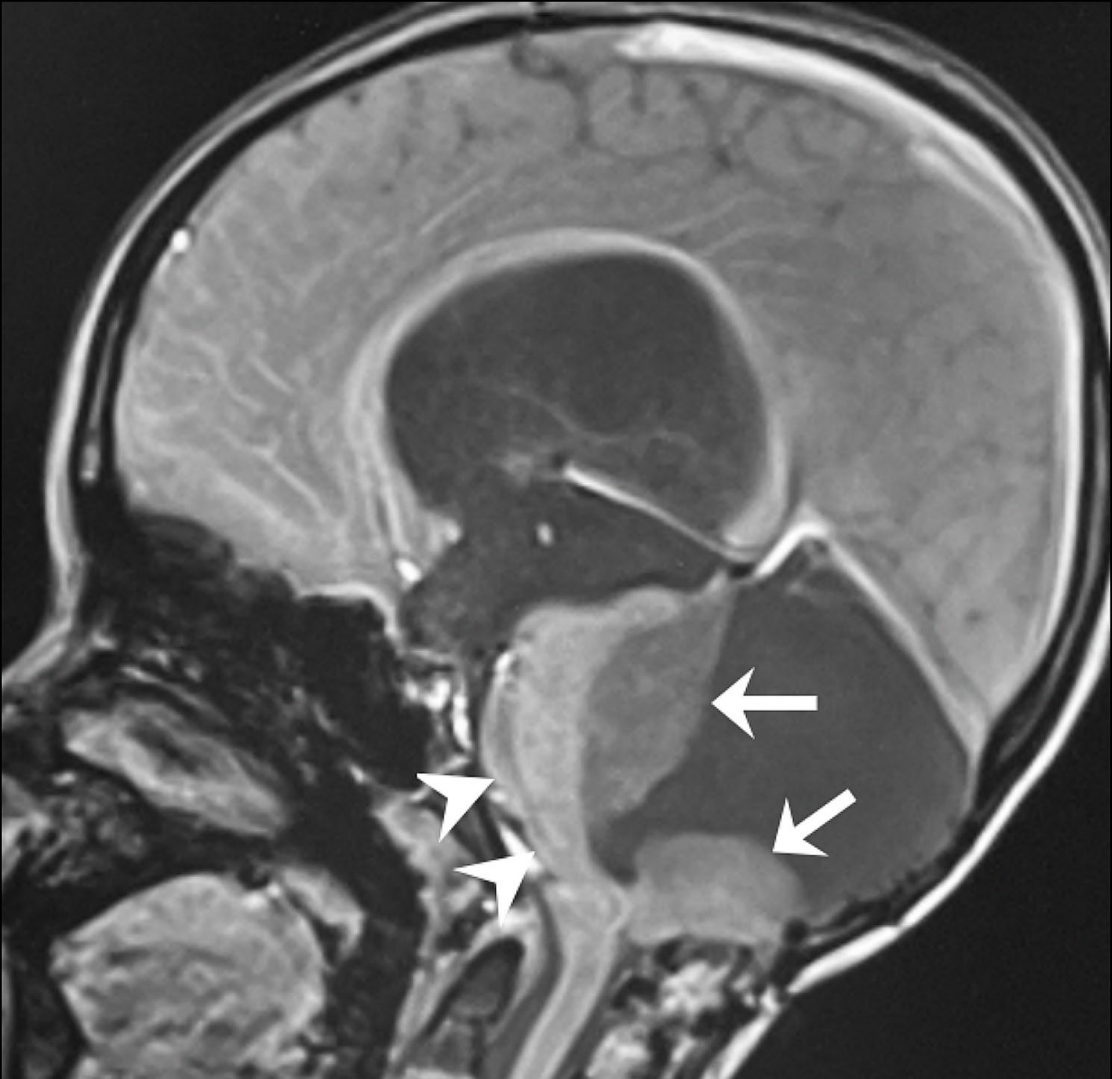
Magnetic resonance images (T1-weighted sagittal section) of brain before surgery. Ten hours before surgery. Arrows and arrowheads indicate cerebellar tumor and brainstem, respectively

In our emergency department, the level of consciousness was I-1 according to the Japan Coma Scale. Body temperature was 36.5℃, pulse rate was 90 bpm (sinus rhythm), blood pressure was 115/78 mmHg, and arterial oxygen saturation was 100% (room air). His pupils were 3 mm in diameter bilaterally, and he showed normal light reflexes. No apparent neurological abnormalities were observed. Magnetic resonance imaging of the brain revealed an enlarged tumor and compression of the brainstem due to enlargement of the fourth ventricle (Fig. 1). He was diagnosed with acute hydrocephalus caused by an enlarged cerebellar tumor, and was urgently admitted. Endoscopic third ventriculostomy and ventriculoperitoneal shunt surgery were scheduled for the next day.

The next morning, just before being taken to the operating room, his level of consciousness deteriorated to Japan Coma Scale III-100 and he became apneic. He was immediately intubated with an uncuffed endotracheal tube (inner diameter 5.0 mm). At that time, his pulse rate and blood pressure were 116 bpm and 90/47 mmHg, respectively. The apnea was considered to be caused by brainstem compression, so he was urgently transferred to the operating room.

**Fig. 2:**
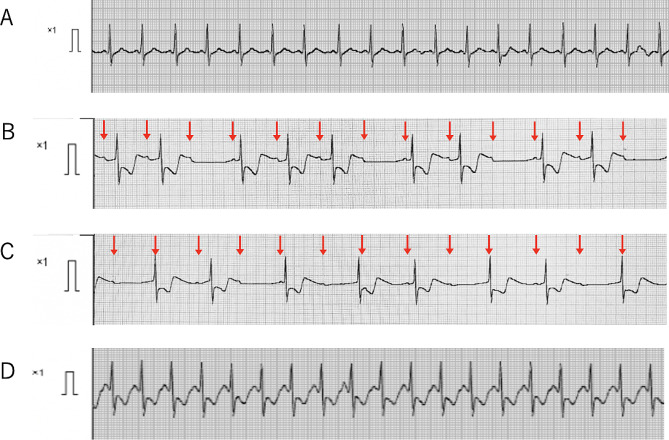
Changes in electrocardiography (ECG) at the time of the first complete atrioventricular block. **A**, ECG recorded at entering the operating room showing sinus rhythm of 102 bpm. **B**, ECG recorded 9 min after administration of remifentanil showing Wenckebach type II AV block, QRS rate of 60 bpm. Arrows indicate P wave. **C**, ECG recorded 13 min after remifentanil administration showing complete AV block, QRS rate of 44 bpm. Arrows indicate P wave. **D**, ECG recorded 5 min after administration of 0.5 mg of atropine sulfate showing sinus rhythm, 140 bpm

**Fig. 3:**
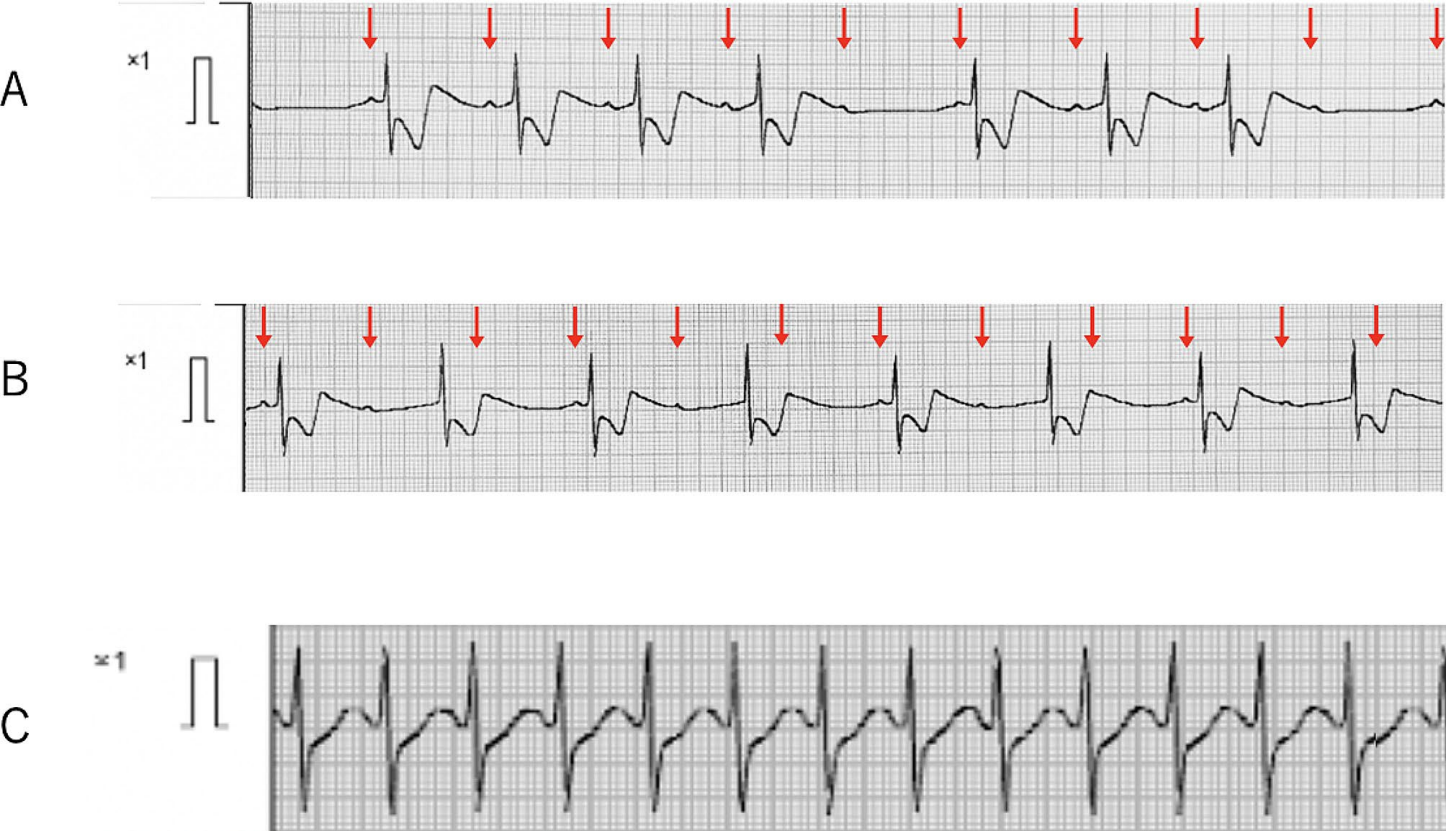
Changes in electrocardiography (ECG) at the time of the second complete atrioventricular block. **A**, ECG recorded 9 min after second remifentanil administration, showing Wenckebach type II AV block with a QRS rate of 75 bpm. Arrows indicate P wave. **B**, ECG recorded 10 min after administration of remifentanil, showing complete AV block, QRS rate of 36 bpm. Arrows indicate P wave. **C**, ECG recorded 8 min after administration of 54 µg of total adrenaline, sinus rhythm was 152 bpm

Electrocardiogram (ECG) showed sinus rhythm with a heart rate of 102 bpm and P-R interval 0.16 msec. Blood pressure was now 94/56 mmHg. Sevoflurane 3% and remifentanil 0.2 µg/kg/min were administered. After obtaining muscle relaxation with rocuronium 1.0 mg/kg, the endotracheal tube was changed to cuffed tube (inner diameter 6.0 mm). Three minutes after starting the remifentanil infusion, the patient’s heart rate decreased from 150 bpm to 100 bpm (Fig. 2A), and 9 min after starting, the pulse rate interval was prolonged from 0.16 s to 0.24 s. ECG showed Wenckebach type II atrioventricular (AV) block (Fig. 2B). Blood pressure was gradually decreasing. Thirteen minutes after starting, Wenckebach type II AV block became complete AV block with heart rate of 44 bpm (Fig. 2B and C). Remifentanil was immediately discontinued, and atropine 0.5 mg was intravenously administered. After that, complete AV block was recovered to sinus rhythm (140 bpm) (Fig. 2D) and blood pressure increased to 102/58 mmHg. Remifentanil (0.2 µg/kg/min) was then resumed. Nine minutes after the infusion, ECG again showed sinoatrial bradycardia, followed by Wenckebach type II AV block (Fig. 3A). Ten minutes after the infusion, Wenckebach type II AV block again became complete AV block with heart rate of 36 bpm (Fig. 3B). Remifentanil was immediately discontinued, adrenaline 4 µg was administered. After administration of additional doses of adrenaline (total 50 µg), complete AV block was recovered to sinus rhythm with heart rate of 152 bpm (Fig. 3C). Thereafter, fentanyl (10–20 µg) was administered intermittently instead of remifentanil, with attention to ECG change during surgery (total dose: 200 µg). The patient’s hemodynamics were stable with continuous dopamine infusion at 5 µg/kg/min, and surgery was successfully completed without bradycardia or AV block. The duration of surgery was 3 h. After surgery, the patient was transferred to the intensive care unit. The endotracheal tube was removed on the second day after surgery. Computed tomography of his brain performed on the fourth day after surgery showed no signs of brainstem compression and there were also no apparent neurological abnormalities. He was discharged from the hospital on the 27th day after surgery. The patient has had no further episodes of bradycardia or AV block.

## Discussion and conclusion

Continuous administration of remifentanil is well known to be a cause of sinus bradycardia, but there are no previous reports of remifentanil-induced complete AV block. In our case, an 8-year-old Japanese boy with acute hydrocephalus caused by brain tumor was shown to have reproducible complete AV block during administration of remifentanil. We discuss the involvement of remifentanil in complete AV block.

Complete AV block during general anesthesia in children can be the result of various factors. Congenital complete AV block is rare and can be found in infants born to mothers with conditions such as Sjogren’s syndrome or systemic lupus erythematosus [4]. In our case, the patient’s mother had no such conditions. Additionally, our patient had no history of loss of consciousness or previous electrocardiogram evidence of conduction disorders. Based on this information, our patient’s complete AV block does not appear to be congenital.

Acquired causes of complete AV block include medication that suppresses the conduction system, surgery of the aortic valve, electrophysiological ablation, and percutaneous coronary intervention [5, 6]. In our patient, there was no history of aortic valve surgery or cardiac catheter therapy. Reproducible complete AV block appeared with remifentanil administration, however, suggesting remifentanil to be the cause in this case of complete atrioventricular block.

Remifentanil has been previously reported as a cause of sinus bradycardia in children [7–9], but there are no known reports of complete AV block. Remifentanil has a negative chronotropic effect due to direct inhibition of the cardiac conduction system and activation of the parasympathetic nervous system [1]. It directly suppresses the sinoatrial node [7]. Meanwhile, there is currently no consensus on the direct effect of remifentanil on the AV node [10, 11]. 

In our patient, after the administration of remifentanil, the pulse pressure was decreased and the pulse rate intervals were gradually prolonged, followed by Wenckebach-type AV block and then complete AV block. These changes, shown by electrocardiogram, can be caused by increased vagal tone [12]. However, because there are no reports of remifentanil causing complete atrioventricular block, we assume that in our case there were other factors in addition to remifentanil that caused vagal activation strong enough to induce AV block. The brainstem was compressed by the tumor (Fig. 1), so the vagal nuclei might have been activated by compression of the tumor or by increased intracranial pressure due to acute hydrocephalus. We therefore speculate that not only the pharmacological action of remifentanil, but also mechanical forces from the tumor and increased intracranial pressure synergistically stimulated the vagal nuclei, which inhibited the AV node and subsequently led to sinus bradycardia, Wenckebach-type AV block, and complete AV block.

In our case, intermittent fentanyl was used instead of continuous remifentanil after recovery from complete AV block. However, it has been reported that there is no significant difference in parasympathetic activity between remifentanil and fentanyl during induction of anesthesia [13]. Therefore, the continuous administration of dopamine and the decrease in intracranial pressure due to surgery may have contributed to prevention of occurrence of complete AV block during the surgery.

In the treatment algorithm for pediatric bradycardia by the American Heart Association, if bradycardia persists, adrenaline at 0.01 mg/kg is given by intravenous injection every 3–5 min, and if bradycardia is suspected to be of vagal origin, atropine at 0.02 mg/kg (minimum dose: 0.1 mg, maximum dose: 0.5 mg, can be repeated once if necessary) is given by intravenous injection [14]. If bradycardia still does not improve, transcutaneous or transvenous pacing should be considered. In our patient, complete AV block was restored to sinus rhythm by administration of adrenaline and discontinuation of remifentanil.

In conclusion, we encountered a case in which the patient with brainstem compression and acute hydrocephalus had reproducible complete AV block during administration of remifentanil. It is possible that the remifentanil augments the vagal reflex caused by increased intracranial pressure, leading to complete AV block.

## Data Availability

The raw data supporting the conclusions of this article will be made available by the author Akihiro Ura (E-mail: akihiro94ura@gmail.com), without undue reservation.

## Abbreviations

ECG: Electrocardiogram
AV: Atrioventricular
